# Behavioral and psychological symptoms in neurodegenerative dementias: harbinger, follower, or constant collateral?

**DOI:** 10.1186/s41983-022-00538-x

**Published:** 2022-09-03

**Authors:** Souvik Dubey, Mahua Jana Dubey, Ritwik Ghosh, Debaleena Mukherjee, Alak Pandit, Julián Benito-León

**Affiliations:** 1 Department of Neuromedicine, Bangur Institute of Neurosciences, Kolkata, West Bengal, India.; 2 Department of Psychiatry, Berhampore Mental Hospital, Murshidabad, India.; 3 Department of General Medicine, Burdwan Medical College, and Hospital, Burdwan, West Bengal, India.; 4 Department of Neurology, University Hospital “12 de Octubre”, Madrid, Spain.; 5 Centro de Investigación Biomédica en Red Sobre Enfermedades Neurodegenerativas (CIBERNED), Madrid, Spain.; 6 Department of Medicine, Complutense University, Madrid, Spain.

**Keywords:** Behavioral symptoms, Dementia, Neuroticism, Obsessive-compulsive disorders, Late-onset behavioral abnormalities, Personality traits, Behavioral biomarkers

## Abstract

Neurodegenerative dementias such as the behavioral variant of frontotemporal dementia, Alzheimer’s disease, and Parkinson’s disease dementia are linked to various behavioral and psychological abnormalities. Whether these abnormalities precede, coincide or follow the onset of cognitive symptoms is still controversial in existing literature, with trajectories available so far dependent on types of dementia. The authors aim to review the different kinds of premorbid behavioral symptoms/personality traits associated with an increased risk of developing specific types of neurodegenerative dementia. Neuroticism has been associated with an increased risk of Alzheimer’s disease and late-onset behavioral abnormalities with the behavioral variant of frontotemporal dementia. The presence of obsessive–compulsive spectrum disorders in Parkinson’s disease dementia is also not rare. Analyzing this evidence, we propose “behavioral biomarkers” as neuroticism in Alzheimer’s disease, late-onset behavioral abnormalities in behavioral variant of frontotemporal dementia, and obsessive–compulsive traits in Parkinson’s disease dementia. These noninvasive behavioral biomarkers will be of immense help, particularly in developing countries, and will prevent the need for costlier investigations and aid in therapeutic strategies.

## Introduction

Neurodegenerative dementias such as the behavioral variant of frontotemporal dementia (bvFTD), Alzheimer’s disease (AD), and Parkinson’s disease dementia (PDD) have been associated with various behavioral and psychological abnormalities. Whether these abnormalities precede, coincide or follow the onset of cognitive symptoms still remains controversial and elusive in existing literature, with trajectories available so far dependent on types of dementia.

The authors, herein, aim to review the different kinds of premorbid behavioral symptoms/personality traits associated with an increased risk of developing specific types of neurodegenerative dementia.

## Behavioral biomarkers: more questions than answers

Behavioral symptoms and dementia have intriguing associations, with undefined temporal relationships concerning the appearance of cognitive symptoms [1]. Behavioral and psychological symptoms of dementia cause a significant negative impact on the health-related quality of life of the patients and their caregivers [1]. The question is whether the premorbid behavioral pattern lies on the same continuum with cognitive problems in dementia. Does it hold true only for patients with Alzheimer’s disease (AD), or can it be extrapolated to other forms of neurodegenerative dementias like frontotemporal dementia or Parkinson’s disease dementia (PDD)?

Most importantly, whether premorbid personality/behavioral patterns switch to different behavioral phenotypes or are merged with cognitive symptoms of dementia is still unresolved. However, several factors (genetics, epigenetics, amyloid deposition, tau deposition, neurotransmitters deficiency/receptors polymorphism, brain networks, and structural damages) underpin the intricate relationship of premorbid behavior symptoms with dementia [2, 3]. Association between premorbid personality/behavior patterns with dementia will potentially help in developing noninvasive, cost-effective and bedside “behavioral biomarker(s)” which, if clinically extrapolated, will be of immense potential help in dementia research, especially in developing countries; not only in prevention and diagnosis, but also in therapeutics.

### Potential behavioral biomarkers for Alzheimer’s disease

AD can be better categorized as a progressive heterogeneous neurodegenerative disorder (with memory deficit being the most prominent symptom) co-clustering with several behavioral symptoms with undefined temporal relationships [4]. Behavioral and psychological symptoms of dementia are present in almost 90% of patients with AD [4]. These symptoms include depression, apathy, anxiety, delusions, hallucinations, agitation, aggression, and disinhibition in the advanced stage [4]. Specifically, the prevalence of depression was 12.7% and 42%, according to the DSM-5 criteria for major depression and the specific criteria for dementia, respectively [5]. Co-clustering of behavioral and psychological symptoms of dementia with subtle cognitive symptoms early in the course of AD often makes the diagnosis difficult [4].

Terracciano et al. [6] reviewed several pieces of evidence decoding the dementia–personality interface and proposed a complex interrelated and integrated interplay between personality traits and AD. Studies depicted that individuals who score higher on conscientiousness (more responsible and self-disciplined) and lower on neuroticism have a reduced risk of developing dementia, even in the presence of AD neuropathology [6-8]. Individuals who score high on neuroticism have a higher risk of dementia [6-8].

The answers to some important questions remain elusive to date: (1) how do personality traits modulate the AD neuropathology?; (2) is there any impact of genetic modulation?; (3) how do premorbid behavior/personality traits determine the onset of cognitive impairment?; (4) do behavioral and psychological symptoms of dementia share its phenotypic similarity with premorbid behavior or whether there is any change?; (5) if there is any change, how and when does it occur? Some studies have unfurled the intricate crucial relationship between premorbid conscientiousness, extraversion, openness, and agreeableness, which proved to be protective factors against behavioral and psychological symptoms of dementia in future [9]. Reverse causation is an upcoming concept in this field of research that can partly explain this phenomenology. Personality changes can be observed in AD neuropathology in the preclinical stage; similarly, increased premorbid neuroticism can adversely modulate AD neuropathology [6-10]. Some studies have unraveled the association between higher neuroticism and advanced spread of neurofibrillary tangles in limbic and neocortical regions. Duron et al. [11] observed that subjects with mild cognitive impairment, high neuroticism, and low conscientiousness scores had a higher white matter burden. This observation has further been substantiated by Booth et al. [12] in a large community-based cohort study. Low conscientiousness was consistently associated with brain-tissue loss, lower white matter fractional anisotropy (a measure of fiber density, axonal diameter, and myelination in white matter), and white matter hyperintensities [12]. Other studies have observed amyloid plaque deposition on scans, mesial temporal atrophy, hypometabolism in the entorhinal region, and neurofibrillary tangles in patients with behavioral symptoms (depression, anxiety, and apathy) who later progressed to overt AD [13].

Taken together, all previous data suggest that behavioral symptoms may be a prelude to AD and also to disease progression.

### Potential behavioral biomarkers for behavioral variant of frontotemporal dementia (bvFTD)

Frontotemporal dementia is subclassified into a few groups: bvFTD, non-fluent variant primary progressive aphasia, and semantic variant primary progressive aphasia and a related group that includes frontotemporal dementia motor neuron disease, progressive supranuclear palsy syndrome, and cortico-basal syndrome [14].

BvFTD is characterized by a diverse spectrum of behavioral problems and personality changes, including psychosis, disinhibition, compulsions, overeating, hyperorality, loss of empathy, and apathy which may appear long before disruption of conventional cognitive domains and thus, may mimic a diagnosis of primary psychiatric disorder [15, 16]. In an extensive systematic review, apathy was the highest reported behavioral symptom in bvFTD (73–100%); anxiety was also frequently reported (19–63%), as well as depression (around 60%) [17, 18].

The neuroanatomical basis of bvFTD has been thought to originate from the dysfunction of cortical brain circuits involving the orbitofrontal cortex (OFC), dorsolateral prefrontal cortex, and medial prefrontal cortex and its subcortical connections (basal ganglia and thalamic structures), primarily responsible for disturbances in emotional states and abnormal social behaviors [15, 16]. Magnetic resonance imaging and 18-fluoro-deoxy-glucose positron emission tomography scan with careful interpretation and proper consideration of genetic background can differentiate between bvFTD and primary psychiatric disorder in patients with late-onset behavioral changes [15].

Patients with bvFTD often show a marked lack of empathy and warmth in interpersonal relationships due to disruption in the right anterior temporal lobe, right medial OFC, and anterior insula [15, 16]. Dysfunction of OFC (right), the supreme higher-order frontal cortical checker of the ‘reward driven’ limbic cortex, results in disinhibition, impulsivity, and socially inappropriate behavior [15, 16]. Repetitive behavior in bvFTD occurs due to frontal subcortical dysfunction [15, 16]. Executive dysfunctions have been claimed to be due to the involvement of the dorsolateral prefrontal cortex [15, 16].

The plethora of psychiatric abnormalities in bvFTD has been associated with the C9ORF72 mutation [19]. Meisler et al. [19] found a proband with bipolar affective disorder harboring C9ORF72 gene expansion, whose father had autopsy-proven frontotemporal lobar degeneration (frontotemporal dementia with different pathological subtypes based on tau, TDP, FUS, and C9ORF72-associated dipeptides), and an earlier diagnosis of bipolar affective disorder. Progranulin (GRN) mutations are associated with a constellation of psychiatric manifestations in frontotemporal dementia, including sexual disinhibition, over-ritualistic behavior, paranoia, and visual hallucinations [20-23]. Diagnosis of schizophrenia and bipolar disorders are common in GRN-mutated frontotemporal dementia patients [20-23]. Contemporary research showed GRN polymorphism is associated with schizophrenia and bipolar disorders, raising issues in favor of a shared pathophysiological basis behind the primary psychiatric disorders and frontotemporal dementia [20-23]. Among considerable uncertainty behind imaging biomarkers of bipolar affective disorder, voxel-based magnetic resonance volumetric studies showed hippocampal volume contractions and subcortical volume changes [24]. Similarly, in schizophrenia, relative grey matter volume deficits in specific frontal brain regions have been found in magnetic resonance voxel-based morphometric studies correlating with earlier age of onset and disease duration [25].

Patients with bvFTD are characterized by grey matter loss and corresponding white matter fractional anisotropy reduction in the frontal and temporal lobe [26, 27]. Diffuse grey matter loss in the frontal lobe (anterior cingulate gyrus) and temporal-limbic substrate correlated well with apathy. In contrast, degeneration/volume loss in temporal-limbic areas and OFC results in disinhibition [26-28]. Though disinhibition is also one of the important behavioral and psychological symptoms of AD, the presence of impulsivity in association with disinhibition favors frontotemporal dementia. [26-28]

### Potential behavioral biomarkers for Parkinson’s disease dementia (PDD)

The clinical characteristic of Parkinson’s disease is not merely confined to motor symptoms as numerous behavioral symptoms such as depression, anxiety, obsessive–compulsive disorders, delusions, hallucinations, psychosis, and cognitive changes are common and manifest at some point in the disease course. The clinical features of PDD encompass cognitive impairment (predominantly executive and visuospatial dysfunctions), behavioral symptoms, autonomic dysfunction, sleep disorders, and parkinsonism [29]. The temporal sequence of appearance of behavioral issues related to cognitive dysfunctions remains elusive in PDD. If there is any specific attribute/predictor of the development of behavioral symptoms in PDD, it is still unexplored. Likewise, any premorbid personality traits/behavior that modulate behavioral symptoms in PDD also remain obscure.

Despite being considered a common neuropsychiatric condition in the general population, the prevalence of the obsessive–compulsive disorder is over 30% in patients with Parkinson’s disease [30]. Though considered two distinct clinical-syndromic complexes, both conditions share a common neuroanatomical and pathological basis, as previously shown in other neuropathological models of dementia [6]. Advanced and sophisticated structural and functional imaging has identified a maladaptive orbitofrontal circuit (circuit connecting the orbitofrontal cortex and basal ganglia) underpinning the pathobiology of obsessive–compulsive disorder [30]. Whether the lack of mental flexibility seen in patients with obsessive–compulsive disorder can be considered an early clinical biomarker for the development of motor rigidity later on seen in Parkinson’s disease patients remains unanswered. There is growing evidence that common neurobiological processes may contribute to vulnerability toward obsessive–compulsive disorder and its persistence in PDD [31-35]. Some studies unveiled abnormalities in the orbitofrontal cortex (volume reduction) and basal ganglia (increase in grey matter volume in globus pallidus and putamen) as most consistent among a wide range of ‘frontostriatal’ loop involvement in obsessive–compulsive disorder [33]. Reduced fractional anisotropy in the corpus callosum and cingulate has been observed in obsessive–compulsive disorder [31-35]. The most convincing evidence of the shared neurobiological basis of obsessive–compulsive disorder and Parkinson’s disease has been demonstrated by Parolari et al. [35]. They showed improvement in the symptoms of obsessive–compulsive disorder and Parkinson’s disease through subthalamic nucleus deep brain stimulation.

Mild cognitive impairment is common in non-demented patients with Parkinson’s disease and is considered a risk factor for dementia [36]. Depression frequently accompanies Parkinson’s disease and is associated with increased disability, rapid progression, mortality, and adverse impact on health-related quality of life [37]. Impulsivity and compulsive behavior in Parkinson’s disease are co-clustered under a common umbrella of impulse control disorder [38]. Pathological gambling, abnormal sexuality/hypersexuality, change in eating pattern (compulsive eating), pathological buying, punding, and hoarding are not infrequent in Parkinson’s disease, claimed to stem from dopamine dysregulation syndrome [38, 39]. Studies revealed the prevalence of impulsivity and compulsive behaviors around 25% among patients with Parkinson’s disease [40]. The dysregulation of the complex interplay of the nigrostriatal, mesocortical, and mesolimbic dopaminergic pathways is responsible for the pathobiological link [36-43].

## Conclusions

From pieces of evidence extrapolated from contemporary research, it can be assumed that behavioral issues and cognitive impairment in various neurodegenerative dementias (i.e., AD, bvFTD, and PDD) are threaded together, sharing some common neuropathological processes (genetics, epigenetics, metabolic, structural, neurotransmitters, receptors polymorphism, pathological and network). The vulnerability and temporal sequence of appearance of behavioral and cognitive symptoms are quite unpredictable and variable, depending on specific types of dementia. The neuropathological basis of developing behavioral issues/personality traits long before overt cognitive symptoms, remains elusive. Still, it is assumed to play in the continuum with the neurobiological basis of the development of cognitive symptoms later on. The complexities of an interrelated and integrated continuum of the neurobiological basis of behavior and cognitive symptoms may not always be evident; however, the basic scientific understanding behind this assumption cannot be ignored. Based on current research depicting the association of high neuroticism and low conscientiousness with AD; the intriguing relationship of late-onset behavioral abnormalities (which is misdiagnosed frequently as late-onset bipolar affective disorder) with bvFTD; and intertwined association between obsessive–compulsive disorder with PDD. Researchers are in a relentless search for developing novel biomarker(s) in neurodegenerative dementias. Although several biomarkers are currently available (advanced imaging biomarkers, genetics, cerebrospinal fluid biomarkers), none proved to be cost-effective and widely available. Consequently, scopes for application of these conventional biomarkers in developing, low and middle income countries are limited.

The authors, herein, propose to consider specific “premorbid behavioral conundrum/personality traits” as a noninvasive, cost-effective, and bedside biomarker(s) of neurodegenerative dementias (high neuroticism in AD, late-onset behavioral abnormalities in bvFTD, and obsessive–compulsive traits in PDD), which will be immensely beneficial, especially in developing low and middle income countries in terms of feasibility of the wider application, cost-effectiveness (in contrast with currently available costlier advanced imaging and cerebrospinal fluid biomarkers) as well as potential prevention (therapeutic modulation of behavioral issues earlier) and earlier diagnosis and treatment of neurodegenerative dementias.

## Abbreviations

AD: Alzheimer’s disease
bvFTD: Behavioral variant of frontotemporal dementia
OFC: Orbitofrontal cortex
PDD: Parkinson’s disease dementia
GRN: Progranulin
